# Autophagy-activating strategies to promote innate defense against mycobacteria

**DOI:** 10.1038/s12276-019-0290-7

**Published:** 2019-12-11

**Authors:** Yi Sak Kim, Prashanta Silwal, Soo Yeon Kim, Tamotsu Yoshimori, Eun-Kyeong Jo

**Affiliations:** 1 Department of Microbiology, Chungnam National University School of Medicine, Daejeon, 35015 Korea; 2 Department of Infection Control Convergence Research Center, Chungnam National University School of Medicine, Daejeon, 35015 Korea; 3 Drug & Disease Target Research Team, Division of Bioconvergence Analysis, Korea Basic Science Institute (KBSI), Cheongju, 28119 South Korea; 4 Department of Genetics, Osaka University, Osaka, 565-0871 Japan; 5 Department of Intracellular Membrane Dynamics, Graduate School of Frontier Biosciences, Osaka University, Osaka, 565-0871 Japan; 6 Department of Medical Science, Chungnam National University School of Medicine, Daejeon, 35015 Korea

**Keywords:** Autophagy, Medical research

## Abstract

*Mycobacterium tuberculosis* (Mtb) is a major causal pathogen of human tuberculosis (TB), which is a serious health burden worldwide. The demand for the development of an innovative therapeutic strategy to treat TB is high due to drug-resistant forms of TB. Autophagy is a cell-autonomous host defense mechanism by which intracytoplasmic cargos can be delivered and then destroyed in lysosomes. Previous studies have reported that autophagy-activating agents and small molecules may be beneficial in restricting intracellular Mtb infection, even with multidrug-resistant Mtb strains. Recent studies have revealed the essential roles of host nuclear receptors (NRs) in the activation of the host defense through antibacterial autophagy against Mtb infection. In particular, we discuss the function of estrogen-related receptor (ERR) α and peroxisome proliferator-activated receptor (PPAR) α in autophagy regulation to improve host defenses against Mtb infection. Despite promising findings relating to the antitubercular effects of various agents, our understanding of the molecular mechanism by which autophagy-activating agents suppress intracellular Mtb in vitro and in vivo is lacking. An improved understanding of the antibacterial autophagic mechanisms in the innate host defense will eventually lead to the development of new therapeutic strategies for human TB.

## Tuberculosis: Helping infected cells take out the trash

Therapies that promote intracellular digestion of microbes could prove a valuable addition to antibiotic weapons against tuberculosis. *Mycobacterium tuberculosis* (Mtb) establishes itself within immune cells, and employs a variety of tricks to protect itself as it sickens its host. Researchers led by Eun-Kyeong Jo at Chungnam National University, Daejeon, South Korea, have reviewed efforts to defeat this pathogen by jump-starting a cellular ‘recycling’ pathway called autophagy. Autophagy helps cells break down both biomolecules aggregates and potential invaders, but Mtb can elude such digestion. Jo and colleagues highlight antimycobacterial agents that can potentially render Mtb vulnerable to autophagy, as well as promising cellular targets that may allow researchers to access this process. For example, evidence suggests that agents that activate a regulatory protein such as ERRα or PPARα could stimulate cellular degradation of Mtb.

## Introduction

There remains a high demand for the development of new drugs against human tuberculosis (TB), which accounts for an estimated 1.3 million deaths globally^1^. TB is mainly caused by *Mycobacterium tuberculosis* (Mtb), a human pathogen that successfully resides in host macrophages and phagocytic cells^2–4^. Macrophages and phagocytes can trigger numerous innate immune signaling pathways, resulting in the activation of effector molecules to combat intracellular parasites, which can exploit host defense strategies through multiple escape mechanisms, leading to the arrest of phagosomal maturation^2,4,5^. Mtb and the host immune system are involved in complicated crosstalk, which requires further investigation. The development of new vaccines and therapeutics against TB requires a comprehensive understanding of the molecular mechanisms underlying the host–pathogen interactions during mycobacterial infection^6,7^.

Autophagy is an intracellular process involved in the housekeeping function and maintenance of cellular homeostasis in response to diverse stress conditions^8,9^. It is becoming clear that the autophagy pathway is vital in the host defense against infection by various intracellular pathogens, including Mtb, *Salmonella enterica serovar* Typhimurium, and *Listeria monocytogenes* through the enhancement of phagolysosome formation^10–15^. This pathway functions as a cell-autonomous defense system that delivers cytoplasmic cargos and bacterial phagosomes for lysosomal degradation^10^. Accumulating evidence has shown that autophagy contributes to innate and adaptive immune pathways in a variety of settings^12,14,16,17^. However, Mtb has evolved numerous strategies to manipulate host innate immune pathways and evade phagosomal acidification^2,18–20^. Furthermore, recent studies have reported that several autophagy genes do not play a critical role in antimycobacterial defense in murine systems in vivo^21^. Nevertheless, numerous drugs/agents are able to induce autophagy activation to promote the restriction and eradication of Mtb in vitro and in vivo^22^. Although there are no specific drugs targeting autophagy, the identification of autophagy-activating small molecules/agents is a promising and new therapeutic target based on host-directed therapy against TB^22–24^. In this review, we present a brief overview of autophagy/xenophagy during Mtb infection and highlight the autophagy-activating agents/molecules that promote host defense against Mtb. We subsequently focus on important recent studies concerning the discovery of new functions of NRs that promote host autophagy and antimicrobial responses against Mtb infection.

## Overview of autophagy in mycobacterial infection

**Fig. 1:**
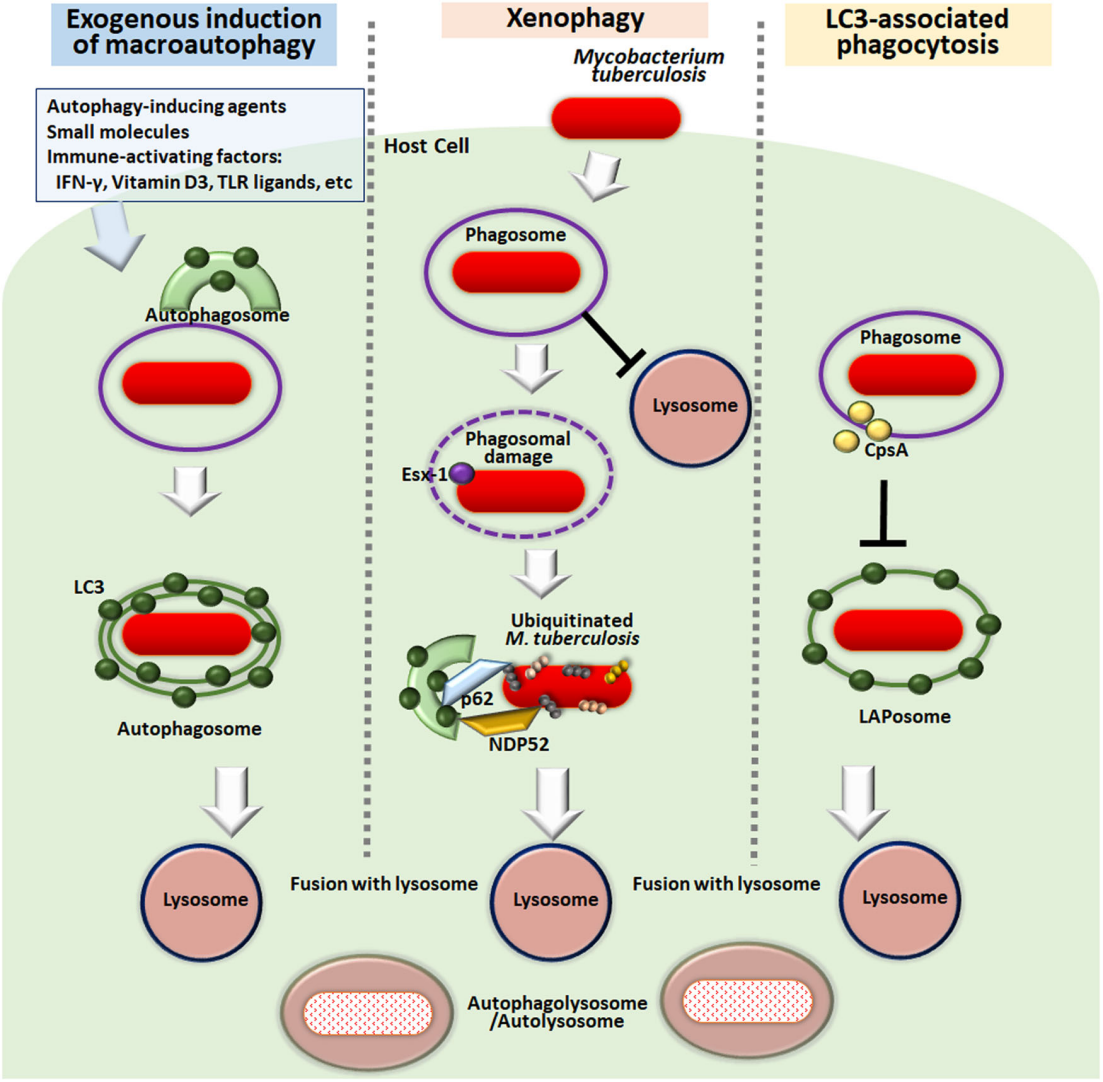
Autophagy pathway activation during Mtb infection. After phagocytosis, Mtb can reside in phagosomes to escape phagosomal acidification. Numerous immunological and pharmacological autophagy activators (box on the *left*) can enhance the restriction of intracellular Mtb growth by overcoming Mtb escape from phagosomal maturation. Mtb phagosome damage through the ESX-1 system is able to trigger ubiquitination of Mtb and its DNA to recruit autophagic adaptors, thereby linking this system to the autophagic machinery. Although much less is known about LAP during Mtb infection, Mtb CpsA has been reported as an inhibitory component in resistance to LAP during infection. IFN interferon, LC3 Microtubule-associated proteins 1A/1B light chain 3B, NDP52 Nuclear domain 10 protein 52, TLR Toll-like receptor

Autophagy (herein, “macroautophagy”) is a multistep process characterized by (1) the initiation of a double-membrane vesicle phagophore; (2) closure as an autophagosome; and (3) fusion with a lysosome to form an autolysosome capable of degrading intracytoplasmic cargo (Fig. 1)^25^. During this process, numerous autophagy-related genes (ATGs), first identified by Dr. Yoshinori Ohsumi^26^, were shown to play essential roles as part of the cellular machinery underlying autophagy^27,28^. In particular, the core machinery of the autophagy process is essential for autophagosome formation. Two ubiquitin-like protein conjugation systems (ATG12 and ATG8/LC3) play critical roles in the formation and ultimate closure of the double-membrane structures of autophagosomes^29^.

Previous studies have shown that the Th1 cytokine Interferon (IFN)-γ activates autophagy in macrophages, leading to an increase in antimicrobial host defense against Mtb infection^12^. Numerous additional studies have reported that activation of macroautophagy can promote phagosomal acidification and antimicrobial responses in murine and human macrophages, suggesting that autophagy may represent a promising host-targeting therapeutic strategy against Mtb infection^22,24,30^. Notably, a recent study by Kimmey et al. showed that ATG5, but no other autophagy genes, plays a unique role in host protection during Mtb infection in mouse models^21^. Interestingly, this protective effect was not mediated through autophagy activation but through the amelioration of excessive inflammatory responses caused by polymorphonuclear neutrophils^21^. These observations suggest that the contribution of individual autophagy genes alone is not sufficient to control the growth of intracellular Mtb. Overall, further investigation is warranted to understand whether activation of autophagy by small molecules and/or compounds could enhance the inhibition of intracellular Mtb replication in vivo.

Once regarded as a simple, nonspecific catabolic process, autophagy has proven far more sophisticated than originally thought and is capable of targeting and degrading specific cellular components, including mitochondria, endoplasmic reticulum, lysosomes, and even invading bacteria^31,32^. Xenophagy is a form of selective autophagy in which cells are able to target and selectively capture bacteria, including Mtb or *Salmonella* Typhimurium, for autophagic degradation^14,28^. Specific processes capable of triggering xenophagy include Mtb phagosomal permeabilization through the ESX-1 secretion system, which can trigger xenophagy activation through ubiquitin-mediated-dependent pathways^13^. Two examples of these pathways are the ubiquitin ligases Parkin and Smurf1, which are involved in the ubiquitination of cytosolic Mtb, followed by its delivery to autophagic machinery^13,33^. The recognition of cytosolic Mtb DNA by the DNA sensor cGAS is required to target Mtb to the ubiquitin-mediated xenophagy pathway^34^. The cGAS-STING pathway is required for type I IFN production, which can compromise host protective immunity against Mtb infection, though the activation of these processes can vary depending on the particular Mtb strain^35,36^. Under most circumstances, the elimination of intracellular Mtb by xenophagy is considered beneficial to the host cells; however, the excessive activation of xenophagy by an Mtb *eis*-deletion mutant induced host cell death and failed to elicit any protective effects in vivo^37^. Taken together, these data suggest that xenophagy activation should be coordinated in conjunction with the appropriate immune responses to promote a more rapid resolution of harmful inflammation, increase cell death and limit the spread of infection.

Another type of noncanonical autophagy pathway is LC3-associated phagocytosis (LAP), which has mainly been studied in fungal infections^38,39^. LAP is an essential link between pattern receptor receptors and phagosomal maturation, helping to enhance the effect of antimicrobial peptides on intracellular pathogens and regulate a variety of physiological functions, including the clearance of apoptotic cells, antigen presentation and type I IFN signaling^40,41^. A recent study found that the Mtb CpsA protein contributes to Mtb escape from the LAP pathway by inhibiting the recruitment of NADPH oxidase 2 (NOX2) to the mycobacterial phagosome^42^. This discovery of the Mtb CpsA protein as a key player in the escape from the LAP pathway has highlighted the need to explore mycobacterial effectors and investigate their ability to modulate canonical and noncanonical autophagic processes during infection^42^. The host autophagy protein Rubicon activates LAP, while inhibiting canonical autophagy^43^. It is necessary to clarify the exact role of Rubicon in autophagy and/or LAP activation during Mtb infection. A schematic overview of autophagy activation during Mtb infection is shown in Fig. 1. In addition, future studies are needed to elucidate the relationship between canonical autophagy and LAP in shaping host protective immune responses during Mtb infection.

## Promotion of antimycobacterial host defense by autophagy-activating drugs/reagents

Mtb and many other pathogens employ numerous strategies to inhibit autophagy^2,19,44,45^. Here, we discuss how the treatment of autophagy-activating agents promotes antimicrobial host defenses in vitro and in vivo by overcoming the ability of bacteria to block xenophagy and dampening excessive inflammation during infection (Table 1).

**Table 1:** Autophagy-activating agents in antimicrobial host defense during mycobacterial infection

Reagent/drug	Class	Mycobacterial species	Experimental model	Mechanism of action	Ref
Rapamycin	mTORC1 complex inhibitor	*M. bovis* BCG, Mtb	RAW264.7 cells, BMDM, and human MDM	Enhancement of mycobacterial phagosome colocalization with LC3, and increases acidification of mycobacterial phagosomes	^12^
Small molecule enhancers of rapamycin (SMER)	mTORC1 complex inhibitor	*M. bovis* BCG	Human PBMC	Induction of autophagy through inhibition of mTOR pathway	^46^
Vitamin D	Vitamin	Mtb	Human monocytes, MDM, THP-1, and RAW 264.7 cells	Increased transcriptional activation of ATG5 and ATG6 through cathelicidin-dependent MAPK and C/EBPβ signaling. Recruitment of cathelicidin to autophagosomes through the Ca^2+^ and AMPK-dependent pathways.	^47^
		Mtb	Human MDM	Cathelicidin LL-37 and autophagic flux activation	^48^
IFN-γ	Cytokine	Mtb	Human T cells, monocytes, MDM, and BMDM	Induction of autophagy and production of cathelicidin via vitamin D-dependent pathway	^49^
		*M. bovis* BCG	RAW264.7, human U937, 293T, and HeLa cells	Induction of autophagy via Irgm1	^50^
Metformin	Antidiabetic drug	*M. bovis* BCG, Mtb	THP-1 cells, human MDM, and mice	Enhancement of mROS production, phagosome-lysosome fusion, and upregulation of lipidated LC3 form	^51^
4-phenylbutyrate (PBA)	Histone deacetylase inhibitor	Mtb	Human MDM and THP-1 cells	LL-37-dependent activation of autophagy by PBA and/or vitamin D	^52^
Nitazoxanide	Antiprotozoal drug	Mtb	Human PBMC, THP-1, MCF-7, MEF, and HEK 293T cells	Inhibition of mTORC1, a negative regulator of autophagy via NQO1	^53^
Fluoxetine	Selective serotonin reuptake inhibitor	Mtb	J774 cells and BMDM	Increased TNF-α production and autophagy Induction	^54^
Gefitinib	EGFR inhibitor	Mtb	J774 cells, BMDM, human MDM, and mice	Autophagy induction and Inhibition of EGFR-mediated p38 activation	^54^
Carbamazepine	Anticonvulsant	*M. bovis* BCG, Mtb, *M. marinum*	RAW264.7 cells, human MDM, alveolar macrophages, zebrafish RAW264.7 cells and mice	mTOR-independent autophagy through IP_3_ depletion and AMPK activation	^55^
Valproic acid	Anticonvulsant	*M. bovis* BCG, Mtb	RAW264.7 cells, human MDM, and alveolar macrophages	mTOR-independent autophagosome formation through ATG12 and inhibition of intracellular bacterial growth	^55^
AICAR	AMPK activator	Mtb, BCG, *M. marinum*	RAW264.7 cells, THP-1 cells, BMDM, mice, and flies	Activation of autophagy through AMPK-PGC1α pathway via C/EBPβ signaling	^56^
		*M. bovis* BCG, Mtb	BMDM, RAW264.7 cells, HEK 293T cells, and mice	ERRα-mediated transcriptional activation of autophagy genes	^57^
Resveratrol	SIRT1 activator	Mtb	BMDM, RAW264.7 cells, HEK 293T cells, and mice	SIRT1-ERRα interaction to activate ATG gene transcription	^57^
		*M. bovis* BCG, Mtb	THP-1 cells and mice	Induction of autophagolysosome in a SIRT1-dependent manner	^58^
SRT1720	SIRT1 activator	*M. bovis* BCG, Mtb	Human MDM, THP-1 cells, and mice	Induction of autophagolysosome in a SIRT1-dependent manner	^58^
Honokiol	SIRT3 activator	Mtb	BMDM, human MDM, and mice	Induction of autophagosome and autophagic flux in a SIRT3-dependent manner	^59^
Isoniazid, Pyrazinamide	Antibiotics	Mtb	BMDM, human MDM, and mice	Autophagy activation by ROS, Ca^2+^, and AMPK-dependent pathway (in Mtb-infected macrophages)	^60^
Loperamide	Anticonvulsant	Mtb	BMDM, murine avleolar macrophages, human avleolar macrophages, MDM, and mice	Increased induction of ATG16L1, LC3 mRNA expression, colocolization of LC3 with Mtb, and reduction of TNF-α production	^61^
Thiostrepton (TSR)	Thiopeptide antibiotic drug	*M. marinum*	RAW264.7 cells and zebrafish	Autophagy activation by endoplasmic reticulum stress pathways	^62^
Statin	Cholesterol-inhibiting drugs	Mtb	Human PBMC, MDM, BMDM, and mice	Reduction of cholesterol levels within phagosomal membranes, promotion of phagosomal maturation and autophagy	^63^
Dehydroepiandrosterone (DHEA)	Steroid hormone	Mtb	THP-1 cells	Induction of autophagosome formation	^64^
Nortriptyline	Anti-depressant	*M. bovis* BCG, Mtb	Human MDM, HeLa cells	Induction of autophagosome formation and autophagy flux	^65^
GW7647, Wy14643	PPARα agonist	*M. bovis* BCG, Mtb	BMDM and mice	Autophagy induction via TFEB, and enhanced lipid catabolism	^66^
GSK4112	NR1D1 agonist	Mtb	THP-1 cells	Increased autophagic flux and TFEB signaling	^67^
Gamma-aminobutyric acid (GABA)	Neurotransmitter	*M. bovis* BCG, Mtb, *M. marinum*	BMDM, RAW 264.7 cells, human MDM, mice, zebrafish, and files	Induces autophagic flux via GABA_A_R, intracellular calcium release, GABARAPL1 induction	^68^

*BMDM* bone marrow-derived macrophages, *MDM* monocyte-derived macrophages, *PBMC* peripheral blood mononuclear cells, *TFEB* transcription factor EB

Previous studies have shown that rapamycin, small molecule enhancers of rapamycin (SMER), vitamin D, interferon-γ, metformin, and 4-phenylbutyrate (PBA) displayed antimicrobial activity against Mtb in human or murine macrophages by enhancing the activation of the autophagy pathway^12,46–52^. In human macrophages, a link between vitamin D-induced autophagy and human cathelicidin microbial peptide (LL-37) has been demonstrated^47,52^. Interestingly, PBA and the active form of vitamin D3 (1,25[OH]_2_D3) were shown to improve intracellular killing of Mtb in human macrophages through LL-37 expression and autophagy^52^.

Several pharmacologic agents have been identified for their ability to induce autophagy to promote antimicrobial effects against Mtb infection. For example, the antiprotozoal drug nitazoxanide and its analogs activate autophagosome formation and mTORC1 inhibition, thus restricting Mtb proliferation in vitro^53^. In addition, a chemical screening study using a high-content microscopic assay identified small molecules that inhibit mycobacterial growth in macrophages by targeting host autophagy activation. It was noted that both fluoxetine (a selective serotonin reuptake inhibitor) and gefitinib (an inhibitor of the epidermal growth factor receptor) activate autophagy and reduce Mtb growth in macrophages and in vivo^54^. Another study with cell-based screening of FDA-approved drugs ascertained that the anticonvulsant carbamazepine and valproic acid enhanced mTOR-independent autophagic killing of Mtb in human macrophages^55^. Recent studies have revealed that AMPK activator (5-Aminoimidazole-4-carboxamide 1-β-D-ribofuranoside, AICAR), sirtuin (SIRT) 1 activator (resveratrol, RSV or SRT1720) or a SIRT3 activator (Honokiol) were beneficial for promoting host defenses against mycobacterial infection through autophagy induction, AMPK activation or reduced inflammation^56–59^.

While host-directed therapy has recently emerged as a new therapeutic strategy for the treatment of human TB, accumulating evidence strongly suggests that antimycobacterial antibiotics exert activities through dual modes, acting on both intracellular bacteria and host autophagy activation^60^. The induction of autophagy by treating macrophages with isoniazid and pyrazinamide was required for successful chemotherapeutic effects against intracellular Mtb. The mechanisms of autophagy activation involved the antibiotic-mediated triggering of hydroxyl radicals and cellular reactive oxygen species in Mtb-infected macrophages^60^. Accumulating evidence suggests that drug repurposing, based on autophagy activation, shows promise in the development of new host-directed therapeutics against Mtb infection. Carbamazepine, loperamide, and valproic acid induce ATG expression and autophagy, which are associated with the control of the intracellular growth of Mtb in murine alveolar cells and alveolar macrophages^61^. Recently, thiostrepton (TSR), a thiopeptide antibiotic possessing a quinaldic acid moiety, has been shown to have a dual action on direct targeting to the bacterial ribosome and the induction of ER stress-mediated autophagy to promote the elimination of intracellular mycobacteria^62^. The cholesterol-lowering drugs, statins showed beneficial effects against intracellular Mtb growth through the promotion of phagosomal maturation and autophagy activation^63^. In addition, the immunomodulatory drug, dehydroepiandrosterone (DHEA) was beneficial in controlling Mtb load through an autophagy mechanism, which contributes to the clearance of Mtb and the prevention of tissue damage^64^. Moreover, the FDA-approved antidepressant drug, nortriptyline can increase autophagosome formation and xenophagic flux against mycobacteria through the synergistic activation of autophagy with IFN-γ^65^. Peroxisome proliferator-activated receptor (PPAR) α agonists (GW7647 and Wy14643) and NR subfamily 1, group D, member 1 (NR1D1) agonist (GSK4112) enhance xenophagic flux via transcription factor EB (TFEB) signaling^66,67^. In our recent report, the major inhibitory neurotransmitter, gamma-aminobutyric acid (GABA) promotes antimicrobial responses and autophagy activation through macrophage type A GABA receptor (GABA_A_R), intracellular calcium release, and the GABA type A receptor-associated protein-like 1^68^. Together, these drugs or agents may act as new therapeutics of host-induced autophagy, thereby enhancing host protection against TB.

## Nuclear receptors and autophagy in mycobacterial infection

NRs are important for innate immune responses to control inflammatory responses and infection^69^. In recent reports, emerging evidence suggests that several NRs play critical roles in autophagy activation to promote the innate host defense against mycobacterial infection. The vitamin D-mediated beneficial effects on the restriction of intracellular Mtb growth in macrophages have been studied; however, additional clinical trials of vitamin D-adjunctive therapies for TB are needed to consider all genetic variants^23,70,71^. NR1D1, an orphan NR, also exerts antimycobacterial effects through the reinforcement of autophagic flux and lysosome biogenesis in human macrophages^67^. We recently showed that orphan NR, estrogen-related receptor α (ERRα; NR3B1, ERR1, ESRRA), promotes macrophage autophagy in response to various autophagy stimulators, including AICAR and RSV^57^. In addition, other studies have reported a role for PPARα in the activation of host defenses in macrophages through autophagy and lysosomal biogenesis^66^. In a recent study of the expression profile of NRs in Mtb-infected macrophages or dendritic cells^72^, several NRs, such as N4a3 and Rora, were identified. Given the findings that numerous NRs appear to be involved in the regulation of autophagy in host cells, future studies are needed to investigate the novel functions of new NRs and their complex interplay with Mtb in the context of autophagy. In this review, we focus on recent studies of the functions of two NRs, ERRα and PPARα.

## ERRα and autophagy

ERRα is the first orphan family member of NRs in which the physiological ligands have not been identified. ERRα, along with other members of the ERRs, does not bind estrogens and preferentially binds to an estrogen-related response element (ERRE) to regulate target genes containing these binding elements in their promoter/enhancer regions^73^. Previous functional studies have shown that ERRα plays a transcriptional activating role through an interaction with the transcriptional coactivator PPARγ coactivator-1α (PGC-1α)^74,75^. ERRα function has been widely studied in the regulation of mitochondrial and metabolic gene transcription, particularly in muscle differentiation, thermogenesis, and in heart and bone functions^76^. Previous works have shown that ERRα is a central regulator of innate immune function, including the regulation of toll-like receptor-induced inflammatory responses and antimicrobial responses against intracellular bacterial infection^77,78^. Recently, a new function of ERRα was revealed in the negative regulation of antiviral responses through the inhibition of type-I interferon signaling^79^.

The involvement of ERRα, in cooperation with PGC-1α, in the mitochondrial quality control and regulation of autophagy has been shown^80^. ERRα deficiency was associated with incomplete autophagy and necrotic cell death in adrenocortical cancer through the control of bioenergetic metabolism^81^. Thyroid hormone induces ERRα, which is essential in the regulation of DRP1-mediated mitochondrial fission and mitophagy through the expression of autophagy-initiating kinase ULK1^82^.

**Fig. 2:**
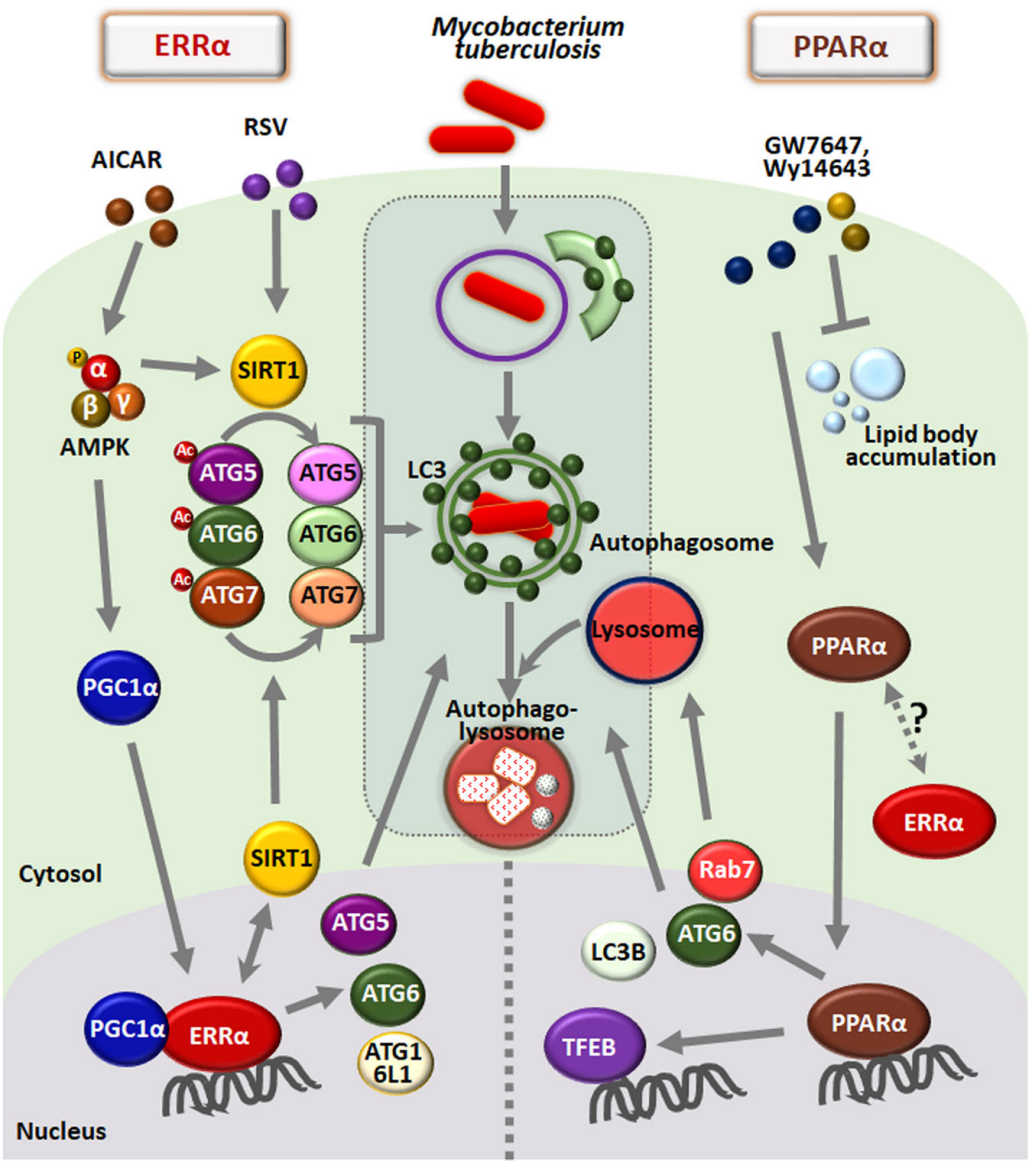
The roles of ERRα and PPARα in autophagy and host defense against Mtb infection. (*Left*) ERRα, which is induced by either AMPK or SIRT1 activation, contributes to the induction of autophagosome formation in BMDMs. ERRα is required for the transcriptional activation of several ATGs containing ERR response elements in the promoters. In addition, the cooperation of ERRα with SIRT1 promotes the deacetylation of ATG5, ATG6, and ATG7, thereby activating autophagy at the posttranslational level. ERRα-mediated autophagy activation results in increased phagosomal maturation and antimicrobial responses during Mtb infection. (*Right*) PPARα, which is activated by PPARα ligands (GW7647 and Wy14643), contributes to enhanced autophagosomal formation and maturation in BMDMs. PPARα is essential for the transcriptional activation of several ATGs, TFEB and lipid catabolism. PPARα reinforces antimicrobial responses to mycobacterial infection by inducing autophagic maturation, TFEB, and lipid catabolism. AICAR, 5-Aminoimidazole-4-carboxamide 1-β-D-ribofuranoside; RSV resveratrol

Notably, ERRα was found to be a key transcriptional regulator of numerous ATGs, including ATG5, ATG6, and ATG16L1, which contain ERR response elements in their promoter/enhancer regions^57^. Although ERRα has no physiological ligands, AMPK and SIRT1 activation enhances the induction of ERRα mRNA and proteins, thereby enhancing the formation of autophagosomes and autophagic flux in macrophages. In addition, ERRα plays a posttranslational regulatory role through the deacetylation of several autophagy proteins, including ATG5, ATG6, and ATG7, all of which are regulated through interactions with SIRT1. Furthermore, ERRα-deficient mice show defective antimicrobial and excessive inflammatory responses against mycobacterial infection, indicating that ERRα is a possible target of antimicrobial innate defenses during Mtb infection^57^. The transcriptional and posttranslational mechanisms by which ERRα regulates the autophagy pathway are shown in Fig. 2.

## PPARα and Autophagy

The NR PPARs include three isoforms (α, δ, and γ)^83^, which form heterodimers with retinoid X receptor and bind to AGGTCANAGGTCA, the peroxisome proliferator response element (PPRE), to induce or repress the transcription of target genes^84,85^. The PPAR target genes are mostly involved in metabolic homeostasis in various tissues, including the liver, adipose tissues, heart and muscle^85–87^. Of the three isoforms of PPARs, PPARα is an important coordinator of lipid metabolism and vascular and inflammatory responses^86,87^. Since PPARα is critically involved in fatty acid oxidation (FAO), lipid and glucose metabolism, and inflammation, the dysregulation of PPARα leads to various defects, such as metabolic, cardiovascular and inflammatory diseases^88–90^. In terms of immunological control, PPARα acts as a critical regulator in immune homeostasis against various inflammatory and infectious stimuli^91–95^. A novel connection between autophagy and PPARα to influence lipid metabolism and innate immunity has been proposed, where autophagy activation by PPARα was shown to promote autophagic lipid degradation and innate host defenses^66^. PPARα activation elevates autophagy, particularly in the transcriptional activation of ATGs^66,96^, which is essential for the regulation of the autophagy process in various tissues and cells^97^. Importantly, there exists a great deal of evidence for crosstalk between PPARα and TFEB^66,98,99^, which is a master regulator of autophagy, lysosomal function and biogenesis, and lipid catabolism^98–100^. Indeed, TFEB is recognized as an important transcriptional factor for the regulation of immune and inflammatory responses^100,101^. Combined with our recent study showing that SIRT3 induces antibacterial autophagy against Mtb infection through PPARα^59^, the function of PPARα in the host defense against intracellular Mtb infection might be primarily mediated through its activation of autophagy^59,66^.

Importantly, a recent report showed that PPARα activation contributes to the enhancement of FAO and lipid catabolism in macrophages during Mtb infection^66^. It would be attractive to examine whether autophagy activation is linked to lipid body inhibition in terms of host defense against Mtb infection. A previous study showed that lipid droplets are delivered to lysosomes via the autophagy pathway, thereby hydrolyzing lipid droplets by the action of lysosomal acid lipase^102^. Thus, autophagy may be required for the regulation of lipid metabolism in macrophages during Mtb infection. PPARα-mediated host defense is summarized in Fig. 2. Gemfibrozil (lipid-lowering drug), an FDA-approved PPARα agonist, has been reported to inhibit the intracellular growth of wild-type and multidrug-resistant Mtb and suppress the activity of enoyl-CoA reductases^103^. For this reason, gemfibrozil may be a potential anti-TB drug candidate; however, it is unclear whether gemfibrozil-mediated antimicrobial responses depend on autophagy activation. It is an open question whether there is crosstalk between PPARα and ERRα in terms of antimycobacterial host defense. Defining the unique immunological features of autophagy-activating agents based on NR function may represent a rational path for designing improved therapeutics or protective vaccines against TB.

## Concluding remarks

Autophagy activation by diverse exogenous stimuli has now been recognized for its role in antimicrobial host defense and in regulating immune and inflammatory responses during Mtb infection. However, the mechanisms controlling these antimicrobial responses are not completely understood. Accumulating evidence shows that autophagy-activating agents are crucial for innate host defense and for controlling excessive inflammatory responses against Mtb infection. Future studies are warranted to examine the effects of autophagy-modulating agents, used either alone or together with chemotherapeutic drugs, for their antimicrobial effects against Mtb infection in vivo and in clinical trials. Given the recent reports showing that both ERRα and PPARα modulate antibacterial autophagy, progress is expected in the development of new therapeutic approaches to treat other infectious diseases beyond tuberculosis. An improved understanding of the molecular mechanisms of autophagy-activating agents will eventually lead to the development of novel therapeutic strategies for human TB.
